# COVID-19 Emotional and Mental Impact on Cancer Patients Receiving Radiotherapy: An Interpretation of Potential Explaining Descriptors

**DOI:** 10.3390/curroncol30010046

**Published:** 2023-01-01

**Authors:** Maria Tolia, Emmanouil K. Symvoulakis, Emmanouil Matalliotakis, Apostolos Kamekis, Marios Adamou, Panteleimon Kountourakis, Davide Mauri, Antonios Dakanalis, Petros Alexidis, Antonios Varveris, Chrysostomos Antoniadis, Dimitris Matthaios, Maria Paraskeva, Constantinos Giaginis, Konstantinos Kamposioras

**Affiliations:** 1 Department of Radiation Oncology, School of Medicine, University of Crete, 71300 Heraklion, Greece; 2 Clinic of Social and Family Medicine, School of Medicine, University of Crete, 71300 Heraklion, Greece; 3 School of Medicine, University of Crete, 71300 Heraklion, Greece; 4 School of Human and Health Sciences, University of Huddersfield, Queensgate, Huddersfield HD1 3DH, UK; 5 Department of Medical Oncology, Mediterranean Hospital of Cyprus, 3117 Limassol, Cyprus; 6 Medical Oncology, University of Ioannina, 45500 Ioannina, Greece; 7 Department of Medicine and Surgery, University of Milano Bicocca, Via Cadore 48, 20900 Monza, Italy; 8 Department of Radiation Oncology, Papageorgiou Hospital, 56429 Thessaloniki, Greece; 9 Oncology Department, General Hospital of Rhodes, 85133 Rhodes, Greece; 10 Department of Food Science and Nutrition, School of Environment, University of the Aegean, Myrina, 81400 Lemnos, Greece; 11 Department of Medical Oncology, The Christie NHS Foundation Trust, Manchester M20 4BX, UK

**Keywords:** COVID-19, emotional, mental health, well-being, cancer, patients, pandemic, health care

## Abstract

Background: Significant changes in the accessibility and viability of health services have been observed during the COVID-19 period, particularly in vulnerable groups such as cancer patients. In this study, we described the impact of radical practice and perceived changes on cancer patients’ mental well-being and investigated potential outcome descriptors. Methods: Generalized anxiety disorder assessment (GAD-7), patient health (PHQ-9), and World Health Organization-five well-being index (WHO-5) questionnaires were used to assess anxiety, depression, and mental well-being. Information on participants, disease baseline information, and COVID-19-related questions were collected, and related explanatory variables were included for statistical analysis. Results: The mean score values for anxiety, depression, and mental well-being were 4.7 ± 5.53, 4.9 ± 6.42, and 72.2 ± 18.53, respectively. GAD-7 and PHQ-9 scores were statistically associated (*p* < 0.001), while high values of GAD-7 and PHQ-9 questionnaires were related to low values of WHO-5 (*p* < 0.001).Using the GAD-7 scale, 16.2% of participants were classified as having mild anxiety (GAD-7 score: 5–9).Mild to more severe anxiety was significantly associated with a history of mental health conditions (*p* = 0.01, OR = 3.74, 95% CI [1.372–10.21]), and stage category (stage III/IV vs. I/II, *p* = 0.01, OR = 3.83, 95% CI [1.38–10.64]. From the participants, 36.2% were considered to have depression (PHQ-9 score ≥ 5). Depression was related with older patients (*p* = 0.05, OR = 1.63, 95% CI [1.16–2.3]), those with previous mental health conditions (*p* = 0.03, OR = 14.24, 95% CI [2.47–81.84]), those concerned about the COVID-19 impact on their cancer treatment (*p* = 0.027, OR = 0.19, 95% CI [0.045–0.82]) or those who felt that COVID-19 pandemic has affected mental health (*p* = 0.013, OR = 3.56, 95% CI [1.30–9.72]). Additionally, most participants (86.7%) had a good well-being score (WHO-5 score ≥ 50). Mental well-being seemed more reduced among stage I–III patients than stage IV patients (*p* = 0.014, OR = 0.12, 95% CI [0.023–0.65]). Conclusion: There is a necessity for comprehensive cancer care improvement. These patients’ main concern related to cancer therapy, yet the group of patients who were mentally affected by the pandemic should be identified and supported.

## 1. Introduction

The World Health Organization (WHO) promptly affirmed the novel coronavirus outbreak as a public health emergency of international concern [1,2,3]. Daily news about the pandemic and its victims showed similarities with cinematographic global disaster narrations [4,5]. At the most critical point of the pandemic, there was no certain prediction on whether the outcome would be positive or negative, hoping for the positive one though [5]. Any dominant scenario or prediction would mostly focus around arithmetic or geometrical death rates [5]. The pandemic had an impact on access to and viability of care services, as well as on health outcomes [6,7,8,9]. There were regions around the world where hospitals were flooded with patients experiencing respiratory symptoms, and this had led the population to deal with the fear of overcrowding and death, as well as burdened access to healthcare [10]. Thus, medical institutions were focused primarily on managing those patients who presented with severe acute disease and infection symptoms [10,11].

Prioritizing COVID-19 patients could lead to potential shortcomings in the care of other vulnerable patient groups, such as cancer patients [12]. Resource allocation has been an ongoing subject of discussion [12,13,14]. A recent systematic review highlighted that even a 4week delay in surgery, systemic therapies, or radiotherapy is associated with a higher risk of death for seven cancer types [15]. Some of the most commonly reported challenges are disruptions of diagnostic and therapeutic procedures, along with timing and type of treatment and modality of care delivery [16,17,18,19,20].There is evidence that radiotherapy was disrupted due to COVID-19. There was a median overall prolongation of the radiotherapy course (52 vs. 44 days) and median break interval (10 vs. 2 days) in COVID-19 head and neck cancer patients. The COVID-RT and non-COVID-RT groups had comparable 1year progression-free rates (84% and 90%, respectively; *p* = 0.08) and overall survival rates at 1 year (86% and 96%, respectively; *p* = 0.06). However, a longer follow-up is warranted [21].

Patients suffering from cancer were among the vulnerable population groups shown to be at a greater risk of severe disease and death from COVID-19. This has been attributed to cancer itself, cancer therapies’ toxicity, and the barriers to cancer care delivery due to the pandemic [8,12,22,23,24,25,26]. In addition, most cancer patients have experienced increased distress as a result of the COVID-19 outbreak [10,27]. An aspect with a potentially negative impact on mental health is social distancing and physical isolation, whether imposed by the government or self-imposed [12,28,29,30,31,32].Cancer patients receiving radiotherapy during the pandemic were reported to have an increased risk of anxiety and depression, requiring emotional support [33]. However, as compared to the pre-COVID-19 period, the emotional burden might not have increased [34,35].

This study evaluated anxiety, depression, and mental well-being levels in cancer patients receiving radiation therapy during the COVID-19 pandemic, as well as descriptors associated with overall service delivery as perceived by participants.

## 2. Materials and Methods

### 2.1. Study Population 

This study included 105 consecutive cancer patients during the COVID-19 pandemic and involved patients receiving radiotherapy for various types of cancer in the Radiation Oncology Department of the University Hospital of Heraklion, in Crete, Greece. Participants were asked to answer a 30 item survey that included questions on their perception of their treatment, risk factors for COVID-19 infection, any previous mental health conditions, and coping strategies used (if any) during the pandemic. All patients participated voluntarily and signed informed consent. The study was performed in compliance with the Helsinki Declaration of 1975, as revised in 2008, and the study protocol was approved by the institution’s review board and conducted following approval by the University General Hospital of Heraklion, Crete, Greece (Protocol No. 18318/21).

### 2.2. Study Measures

We used the validated self-reported Generalized Anxiety Disorder scale (GAD-7), which is useful and effective in primary care and mental health settings as a screening tool and severity measure for anxiety symptoms [36]. GAD-7 is a 7item scale covering the period of the past two weeks, with items rated on a 4point Likert scale: “not at all” (0 points), “on individual days” (1 point), “more than half the days” (2 points), or “nearly every day” (3 points). The sum of these items gives the final GAD-7 score, ranging from 0 to 21, with higher values indicating more severe anxiety symptoms. We also used the validated Patient Health Questionnaire 9 (PHQ-9), which is a self-administered version of the PRIME-MD diagnostic instrument for common mental disorders [37]. The PHQ-9 is the depression module, which scores each of the 9 DSM-IV criteria from “0” (not at all) to “3” (nearly every day). The PHQ-9 score can range from 0 to 27, with higher values indicating more severe symptoms of depression [37]. Moreover, the World Health Organization-Five Well-Being Index (WHO-5) was used [38,39]. The WHO-5 is a short, self-reported measure of current mental well-being [39]. Each of the 5 items is scored from 5 (all the time) to 0 (at no time), with the raw score ranging from 0 (absence of well-being) to 25 (maximal well-being). Since scales measuring health-related quality of life are conventionally translated to a percentage scale from 0 (absent) to 100 (maximal), it is recommended to multiply the WHO-5raw score by 4 [39].

### 2.3. Design-Outcomes

The goal of the study was to evaluate the psycho-emotional impact of COVID-19 on patients participating in the survey. Population baseline characteristics (e.g., age, sex, family status, primary tumor, stage, etc.), disease monitoring data, and COVID-19-related questions were recorded and included in the statistical analysis. The study’s endpoints were anxiety, depression, and mental well-being, which were evaluated using the GAD-7, PHQ-9, and WHO-5 questionnaires, respectively (see also the statistical analysis). The treating physician was responsible for informing the patients about the purpose of the study, explaining the procedures, obtaining signed informed consent, distributing the self-administered questionnaires, and collecting the data. All questionnaires were answered during the course of radiotherapy.

### 2.4. Statistical Analysis

Descriptive statistics were used to analyze the cohort baseline features and the data collected from the COVID-19-related questions. Those included were means with standard deviation (SD) or medians with interquartile range (IQR) according to the normality assumption for continuous variables, while counts with percentages (*n*, %) were presented for categorical variables. Univariate and multivariable logistic regression models were undertaken to identify associations between descriptors and the outcomes of interest. A Spearman’s rank correlation coefficient test was used to investigate correlations among the evaluation scales. In line with prior studies [39,40,41,42,43], the results from the self-reported questionnaires were dichotomized using two different cut-off values at 5 (score ≥ 5 indicating mild level) and 10 (score ≥ 10 indicating moderate and more severe level) for the GAD-7 and PHQ-9 scales, and 50 for the WHO-5 scale, as described above. Level groups were analyzed as binary variables, using logistic regression models to investigate potential associations between covariates and the outcomes of interest. The explanatory variables included in the multivariable analysis were those with a *p*-value in univariate comparison ≤ 0.15. In the multivariable analysis, the goodness of fit for the regression models was checked by the Hosmer–Leeshawn test. The odds ratios for each predictor variable in the final model, along with their 95% CIs and *p*-values, were presented. The level of statistical significance was set at 0.05 for all tests. SPSS-25 software was used for the statistical analysis.

## 3. Results

### 3.1. Survey Participants

During a six month period in 2021, 105 consecutive patients who underwent radiotherapy were invited to participate in the study. All participants had no treatment delays or deferrals due to the COVID-19 pandemic. Population baseline characteristics and COVID-19-related questions are outlined in Table 1. Most of them were patients in stage I (*n* = 30) and stage III (*n* = 33). Additionally, most participants were female (*n* = 64, 61%) and in the age group 61–70 years (*n* = 31, 29.5%). The most common malignancy was breast cancer (*n* = 43; 41%), followed by lung cancer (*n* = 17; 16.2%), and prostate cancer (*n* = 10; 9.5%). Most participants lived in a family environment since only 10% answered that they were single and 24.8% lived alone. Almost half of the participants stated they had suffered from a mental health complaint in the past that could affect their psychological status during the pandemic, with anxiety, panic attacks, and depressive symptoms being the most prevalent. One third of the participants suffered from at least one medical condition that affected the risk of severe infection, not the risk of being infected with COVID-19.More specifically, 37.3% had a history of chronic lung disease, 27.1% had been diagnosed with diabetes, and 30.5% were submitted to immunosuppressive therapies.

### 3.2. Exposure to COVID-19

In our study, most of the participants (*n* = 98, 93.3%) wished to have a COVID-19 test before their cancer treatment, and only six patients tested positive and needed to be admitted (Table 2). Most of them (80%) were more worried about their cancer than COVID-19, while 17.1% and 48.6% were “not at all” and “slightly” concerned about getting infected, respectively. However, a significant proportion (*n* = 46; 43.8%) were worried that COVID-19 could have a negative impact on their cancer treatment (Table 2).

### 3.3. Coping and Support Mechanisms

Participants reported various coping mechanisms used during the period of the pandemic (Table 1). Change in physical activity (46.7%), positive attitude (36.2%), and time management (30.5%) were widely used choices. Less than one third of the participants (26.7%) chose to talk to medical professionals as a personal coping strategy. Nevertheless, a specialist nurse (82.9%) and cancer team (76.2%) were reported to offer a lot of support during this distressing period. Community services and government initiatives were scored as low, with 70.5% and 80% stating that they were not at all to slightly satisfied, respectively. Patients felt that friends/family (87.6%) and their general practitioner (81.9%) offered them the greatest support.

### 3.4. Correlation between Scores of the Questionnaire GAD-7, PHQ-9, and WHO-5 

The GAD-7, PHQ-9, and WHO-5 mean scores were 4.7 (SD = 5.53), 4.9 (SD = 6.42), and 72.2 (SD = 18.53), respectively. A Spearman’s rank correlation coefficient test showed a statistically significant correlation between GAD-7 and PHQ-9 (r = 0.596; *p* < 0.001), while inverse relationships were observed between WHO-5 and GAD-7 (r = −0.494; *p* < 0.001) and between WHO-5 and PHQ-9 scores (r = −0.528, *p* < 0.001).

### 3.5. Assessing the Emotional and Mental Health Impact of COVID-19

**Figure 1:**
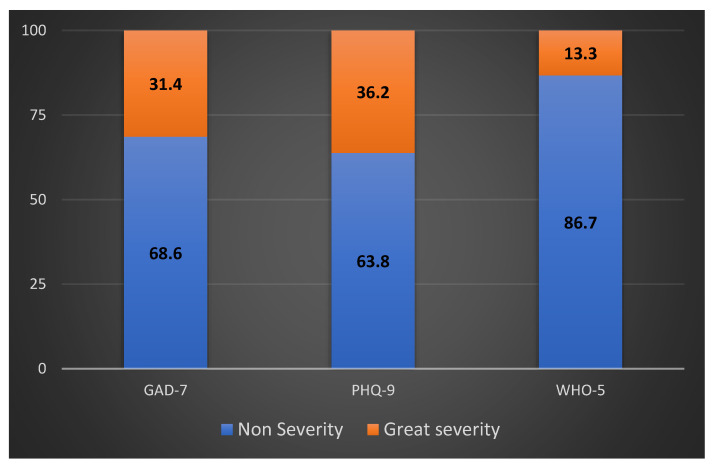
Frequency distribution (%) of GAD-7, PHQ-9, and WHO-5 scores among enrolled participants. “Non-severity” includes scores within normal limits. “Great severity” includes scores at pathological limits, regardless of severity grade.

All 105 participants completed the questionnaires. Inferentially, 68.6% of participants do not have anxiety (GAD-7 score, <5), 16.2% have mild anxiety according to the GAD-7 scale (GAD-7 score, 5–9), and 15.2% have moderate or more severe anxiety (GAD-7 score ≥ 10) (Figure 1). After the Hosmer–Leme show test, anxiety levels were correlated with those who had a previous diagnosis of a mental health condition and those who had advanced disease stages (*p* < 0.001). These parameters were significantly associated with a higher risk of anxiety in multivariable analysis (OR = 3.74, 95% CI: 1.37–10.21; *p* = 0.01; and OR = 3.83, 95% CI: 1.38–10.64; *p* = 0.01) (Table 3).

From the participants, 63.8% were considered normal or to have minimal depression (PHQ-9 score < 10), with 20% having moderate or more severe depression (PHQ-9 score ≥ 10) (Figure 1). In multivariable analysis, older patients (OR = 1.63, 95% CI: 1.16–2.30; *p* = 0.05), patients with previous mental health conditions (OR = 14.24; 95% CI: 2.47–81.84; *p* = 0.03), patients concerned that COVID-19 would have a negative impact on the cancer treatment (OR = 0.19; 95% CI: 0.045–0.82; *p* = 0.027), and those who felt that the COVID-19 pandemic had affected mental health (OR = 3.56, 95% CI: 1.30–9.72; *p* = 0.013) were more likely to have moderate or more severe depression (Table 3). 

Most participants (86.7%) had good well-being (WHO-5 score ≥ 50) (Figure 1). In multivariable analysis, stage I–III patients were more likely to have reduced mental well-being compared to stage IV (*p* = 0.014, OR = 0.12, 95% CI [0.02–0.65]) (Table 3).

## 4. Discussion

Daily life has been affected by the COVID-19 pandemic in many ways, and this has also had a significant impact on medical practice. The general population was experiencing an increasing amount of emotional and physical pressure, with uncertainties in public health and severe limitations on social life affecting their mental health and psychological resilience [10,30]. For cancer patients, diagnosis, treatment, and follow-up are generally related to increased levels of distress [10,40]. The same patients have been significantly affected by the COVID-19 outbreak, given that cancer treatments are typically time-sensitive and personalized, and the nature of the disease does not allow for degrees of flexibility [41]. As part of this study, we measured the psychological impact of the pandemic on people receiving cancer treatment by evaluating their levels of anxiety and depression, as well as their overall well-being.

Most of the patients participating in the survey were not really concerned about getting a coronavirus infection. Most reported that they were “slightly” or “not at all” concerned about being infected, and eight out of ten reported that they were more concerned about their disease than COVID-19 infection. This reflects the fact that the continuation of cancer management remains the main concern among these patients. A related survey presented quite similar findings, supporting alternative methods of consultation, such as telephone or video calls, as a means of creating a safe environment for patients during infection outbreaks without compromising medical care continuity [42]. In addition, the importance of delivering optimal cancer treatment has been previously highlighted, as it has been reported that the risk of death from COVID-19 in cancer patients receiving therapy was not different from that of those not receiving treatment [42,43]. The treatment should thus continue as indicated, as long as all the appropriate safety measures against COVID-19 are taken.

Patients participating in our survey reported that family/friends, the cancer team, a specialist nurse, and their general practitioner offered them the highest level of support, whereas community services and government initiatives received lower scores. The importance of family environment in managing stress has already been described [44], and the debated results regarding government initiatives and community services should be noted for future health planning communications. It is possible that patients’ expectations have not been met, and this could be subject to further investigation in the future. Additionally, support efforts for patients may not have been sufficiently communicated to the public. The public is not a whole, as it is usually approached, and many sub-groups with different needs and expectations compose a human “mosaic”. According to a previous study, half of the patients participating were not aware of the relevant services and were often confused by the advice given or the messages received [43]. As far as our findings are concerned, further effort is potentially needed to reach out to patients as well as understand and satisfy their need for support.

Participants stated they found the cancer team very supportive, and one in four referred to medical professionals for support, although this was not the most popular coping attitude. Different coping mechanisms were used, with 46.7% preferring to use physical activity, which was among the most popular. It was also interesting to notice that one third of the participants used different coping mechanisms from the ones suggested in our survey, and it would be useful to explore them in a subsequent survey.

The most common descriptor of the outcomes of interest was underlying mental health conditions, significantly associated with mild to more severe anxiety and depression. Our findings highlight the importance of mental health status, and it is likely that certain groups of patients needed greater support during the pandemic or similar stressful conditions. The association of more severe depression with the fear that COVID-19 will negatively affect treatment could be due to the fact that alternative methods of consultation or treatment were perceived by patients as degrading the medical services provided. It is not uncommon for patients receiving cancer treatments to be uneasy about treatment postponement, deferrals, or alterations in treatment strategies [44,45,46,47]. This highlights the need for more efficient and comprehensive information by the primary care sector about the new collaborative strategies with the public health sector, introduced during periods most in need [48].

### Strengths and Limitations

None of the endpoints investigated in our survey were associated with conditions relevant to COVID-19, with the exception of more severe depression. This suggests that the pandemic and the relevant restrictive measures or changes in medical practice did not have a dramatic impact on the patients’ psychological status, since this status already deserved attention due to the nature of the disease. The questionnaires were completed in the presence of doctors, and this was welcome as they could clarify, in a structured and previously informed manner, any potential questions that patients had. The small sample size and the fact that patients were recruited from a single hospital center were both limitations of this study. We also cannot estimate what the net effect of radiotherapy is on emotional or mental health status and how these vary during the progression of the sessions. To what extent the type of cancer can be treated is also a source of complexity and needs careful research design. Additionally, this study was conducted during the COVID-19 pandemic, which may have put a collective strain on patients’ mental well-being with the many gaps that emerge between rational and perceived conceptualizations. The COVID-19 stress questionnaire was developed for crude data collection in order to capture easy responsiveness meanings without burdening patients waiting for their session. This could also be deemed a limitation, as only basic socio-demographic information was collected. Possibly relevant information regarding socioeconomic status, race, ethnicity, cultural background, living environment, and religious activity was missed. The causal root of an oncogenic process and the nature of factors influencing exposure are parameters to be considered when research variables are chosen or set [49]. We therefore cannot assess the influence of the previously mentioned possible confounding factors.

## 5. Conclusions

According to the findings of this survey, patients felt that relatives, members of multidisciplinary cancer health teams, and their general practitioner (GP) offered them a greater sense of safety in comparison to government- and community-driven initiatives. Moreover, it is reassuring that the frequency of participants with moderate to severe anxiety was as low as expected. Importantly, patients were much more concerned about their cancer treatment than about COVID-19, which emphasizes the necessity to continue to provide comprehensive cancer care even in the case of the persistence of COVID-19 in the community.

Additionally, further research on the impact of the COVID-19 pandemic on both physical and mental health is recommended, but it is important to continue research on the medical and social environment in order to identify inequalities, gaps, and deficiencies in vulnerable groups such as cancer patients. Providing appropriate psychological support to those in need and giving intelligible information is a duty. A suggestion of our study to health care service providers and policymakers is the necessity to evaluate and monitor the medical needs of cancer patients, with an emphasis on the emotional and social well-being of these patients.

## Figures and Tables

**Table 1:** Descriptive characteristics of 105 participants in the current study.

Sex	Male	41	39.0%	Primary Tumor	Breast	43	41%
	female	64	61.0%		Lung	17	16.2%
Age, years	<50	30	28.6%		Prostate	10	9.5%
	51–60	18	17.1%		Endometrium	9	8.6%
	61–70	31	29.5%		Rectum	6	5.7%
	>70	26	24.8%		Cervix	5	4.8%
Family status	married/in civil partnership	68	64.8%		Larynx	4	3.8%
	unmarried, divorced, widowed, in relationship	37	35.3%		Brain	2	1.9%
Child existence		91	86.7%		Anum	2	1.9%
Single-person household	no	79	75.2%		Bladder	1	1.0%
Previous diagnosis of mental disorder/s *	Anxiety	37	35.2%		Esophagus	1	1.0%
	Panic attacks	25	23.8%		Nasal Cavity	1	1.0%
	Depression	14	13.3%		Pancreas	1	1.0%
	Anorexia	9	8.6%		Parotid	1	1.0%
	Bulimia	7	6.7%		Thymoma	1	1.0%
	Obsessive-compulsive disorder	5	4.8%	Stage	I	30	28.6%
	Social Phobia/Stress	4	3.8%		II	26	24.8%
	Psychosis	2	1.9%		III	33	31.4%
	Attention deficit disorder	2	1.9%		IV	16	15.2%
	Alcohol/Drug Abuse	1	1.0%	Conditions	Severe chronic obstructive pulmonary disease (e.g., COPD, bronchitis, cystic fibrosis)	22	37.3%
No previous diagnosis of mental disorder/s *		53	50.5%		Immunosuppressive treatment	18	30.5%
Self-reported perception of the current status of cancer	No active disease or under control	56	54.4%		Diabetes Mellitus	16	27.1%
	Progressive disease	6	5.8%		Obesity	2	3.4%
	In progress, unknown, or other	41	39.8%		Transplantation in the past (e.g., heart, kidney, bone marrow, etc.)	1	1.7%
					Pregnancy	1	1.7%
					Change in physical activity (e.g., exercise)	49	46.7%
				Coping mechanisms used during the pandemic	Positive attitude	38	36.2%
					Time management	32	30.5%
					Overlook	28	26.7%
					Discuss with medical professionals	28	26.7%
					Distract himself/herself	26	24.8%
					Make use of humor	26	24.8%
					Changes in diet (e.g., types of food, amount)	16	15.2%
					Utilize religious or spiritual practice(s)	11	10.5%
					Utilize meditation, mindfulness, or other relaxation techniques	7	6.7%
					Change in substance intake (e.g., smoking, alcohol, other drugs)	5	4.8%
					Other	1	1.0%
					None of the above	36	34.3%

* Participants might have stated more than one mental condition.

**Table 2:** Responsiveness to items related to the COVID-19 exposure.

COVID-19-Related Questions	Response	N	%
Testing for COVID-19	Yes	104	99.0%
	No	1	1.0%
Testing positive	Yes	6	5.7%
	No	99	94.3%
Willingness to be tested, before anticancer treatment	Yes	98	93.3%
	No	7	6.7%
Hospitalization for COVID-19	Yes	6	5.7%
	No	99	94.3%
Concern about the negative impact of COVID-19 on cancer therapy	Yes	46	43.8%
Greater concern about COVID-19 than cancer	Yes	21	20.0%
	No	84	80.0%
Concern for COVID-19 contraction	Not at all	18	17.1%
	Slightly	51	48.6%
	Moderately	18	17.1%
	Very much	17	16.2%
	Extremely	1	1.0%

**Table 3:** Multivariable analysis of variables tested associated with GAD-7, PHQ-9, and WHO-5.

Scales	Variables	Odds Ratio (95% CI)	*p*-Value
Anxiety (GAD-7 ≥ 5)	Previous/Underlying diagnosis of a mental health condition (“No” vs. “Yes”)	3.743 (1.372–10.210)	0.010
	Felt the COVID-19 pandemic has affected mental health	1.841 (0.839–4.041)	0.128
	Needing more support for mental health during COVID-19	0.422 (0.126–1.420)	0.163
	Concern about getting COVID-19	0.977 (0.446–2.141)	0.953
	Stage category (Stage I/II vs. III/IV)	3.832 (1.380–10.640)	0.010
Depression (PHQ-9 ≥ 10)	Age	1.637 (1.163–2.305)	0.005
	Previous/Underlying diagnosis of a mental health condition (“No” vs. “Yes”)	14.242 (2.478–81.847)	0.003
	Concerned that COVID-19 had/will have a negative impact on their cancer treatment (“No” vs. “Yes”)	0.193 (0.045–0.828)	0.027
	Concern about getting COVID-19	1.145 (0.433–3.033)	0.785
	Felt the COVID-19 pandemic has affected mental health	3.561 (1.304–9.726)	0.013
	Needing more support for mental health during COVID-19	1.359 (0.262–7.059)	0.715
Well-being (WHO-5 ≥ 50)	Previous/Underlying diagnosis of a mental health condition (“No” vs. “Yes”)	0.278 (0.056–1.366)	0.118
	Self-reported perception of the current status of cancer	0.561 (0.117–2.691)	0.470
	Concerned that COVID-19 had/will have a negative impact on their cancer treatment (“No” vs. “Yes”)	1.827 (0.404–8.252)	0.434
	Concern about getting COVID-19	0.561 (0.188–1.677)	0.301
	Felt the COVID-19 pandemic has affected mental health	0.876 (0.318–2.411)	0.797
	Needing more support for mental health during COVID-19	1.086 (0.176–6.707)	0.929
	Stage category (Stage IV vs. other)	0.121 (0.023–0.651)	0.014

## Data Availability

The data presented in this study are available in this article.
